# Antibody Responses in Humans Infected with Newly Emerging Strains of West Nile Virus in Europe

**DOI:** 10.1371/journal.pone.0066507

**Published:** 2013-06-12

**Authors:** Stefan Chabierski, Gustavo R. Makert, Alexandra Kerzhner, Luisa Barzon, Petra Fiebig, Uwe G. Liebert, Anna Papa, Justin M. Richner, Matthias Niedrig, Michael S. Diamond, Giorgio Palù, Sebastian Ulbert

**Affiliations:** 1 Department of Immunology, Fraunhofer Institute for Cell Therapy and Immunology, Leipzig, Germany; 2 Department of Molecular Medicine, University of Padova, Padova, Italy; 3 Institute of Virology, Leipzig University, Leipzig, Germany; 4 Department of Microbiology, Medical School, Aristotle University of Thessaloniki, Thessaloniki, Greece; 5 Departments of Medicine, Molecular Microbiology, Pathology & Immunology, Washington University School of Medicine, Missouri, St. Louis, United States of America; 6 Robert Koch Institute, Berlin, Germany; German Primate Center, Germany

## Abstract

Infection with West Nile Virus (WNV) affects an increasing number of countries worldwide. Although most human infections result in no or mild flu-like symptoms, the elderly and those with a weakened immune system are at higher risk for developing severe neurological disease. Since its introduction into North America in 1999, WNV has spread across the continental United States and caused annual outbreaks with a total of 36,000 documented clinical cases and ∼1,500 deaths. In recent years, outbreaks of neuroinvasive disease also have been reported in Europe. The WNV strains isolated during these outbreaks differ from those in North America, as sequencing has revealed that distinct phylogenetic lineages of WNV concurrently circulate in Europe, which has potential implications for the development of vaccines, therapeutics, and diagnostic tests. Here, we studied the human antibody response to European WNV strains responsible for outbreaks in Italy and Greece in 2010, caused by lineage 1 and 2 strains, respectively. The WNV structural proteins were expressed as a series of overlapping fragments fused to a carrier-protein, and binding of IgG in sera from infected persons was analyzed. The results demonstrate that, although the humoral immune response to WNV in humans is heterogeneous, several dominant peptides are recognized.

## Introduction

West Nile Virus (WNV) is a mosquito-borne flavivirus that was first described in Africa in 1937 and is now endemic to large parts of the tropical and subtropical world. In its natural cycle, WNV cycles between mosquitoes and birds but also can be transmitted to and cause infection and disease in humans and other vertebrate animal species. Clinical manifestations of WNV infections in humans range from no symptoms, to a febrile syndrome, to neuroinvasive disease including meningitis, encephalitis, and acute flaccid paralysis. The most severe neuroinvasive forms affect ∼1% of the WNV-infected humans, primarily the elderly or immunocompromised [1]. The virus reached public attention when it was introduced into North America in 1999, and since has spread over the entire continent [2]. Since the mid-1990s, WNV has emerged in both Eastern and Western Europe, where before it only sporadically caused mild or limited local outbreaks. In comparison, over the last few years, outbreaks with severe disease have been reported in Romania, Hungary, Russia, Italy, Former Yugoslav Republic of Macedonia, and Greece, where the virus is now considered endemic [3]–[6]. As an example, almost 200 severe WNV infections with 33 deaths were reported in Greece in 2010 [7]. The rate of severe neurologic disease was similar to the one observed in the USA [AP, unpublished].

The positive-stranded RNA genome of WNV encodes for the three structural proteins capsid (C), pre-membrane/membrane (prM/M) and the envelope (E) protein, as well as seven non-structural proteins [8]. The C protein lies within the inner core of the virus and binds to viral RNA to form the nucleocapsid. E is the major surface glycoprotein on the mature virion and functions in several critical events during the viral life cycle, including receptor binding, entry, and endosomal fusion. The small glycoprotein M (and its precursor prM) acts as a chaperone for E protein folding and also is displayed on the virion surface, albeit in a location proximal to the viral membrane [9]. Most WNV isolates are classified into two major lineages, termed lineage 1 and 2, which share ∼75% identity at the nucleotide and ∼94% at the amino acid level [10]. Unlike the epidemiology in the Americas, where a lineage 1 WNV strain is exclusively detected, there are several strains belonging to different lineages that circulate in Europe. Co-circulation in the same area of different WNV strains belonging to both lineage 1 (clade 1a) and lineage 2 has been reported in Italy [3], [11]–[13], and different WNV lineage 2 strains were responsible for outbreaks in Greece, Romania, and Russia [4], [14]. Co-circulation of WNV of different lineages must be considered for the development of vaccines, therapeutics, and diagnostics.

The humoral immune response to a WNV infection in small animal models is characterized by the appearance of IgM antibodies after 4 to 7 days, with antigen-specific IgG detectable shortly after [15]. Both neutralizing IgM and IgG are important means for controlling the infection and likely limit the viremia that results in WNV dissemination into the central nervous system [16], [17]. Antibodies against WNV are also detected by immunoassays to diagnose infections, however, cross-reactivity with other circulating flaviviruses can confound diagnosis; thus, functional assays, such as a neutralization test, or nucleic acid based detection systems are required to confirm a diagnosis of WNV infection [18]–[20].

The human immune response to American WNV infections is being studied extensively. Epitopes for CD8^+^ T cells and for several human monoclonal antibodies have been mapped [21]–[24]. B cell epitopes also have been identified by screening selected WNV proteins with sera from birds, rodents and horses, but only to a limited extent with sera from infected humans [25]–[28]. In addition, only few data exist as to the immune responses to newly emerging European WNV strains.

Herein, we describe a platform to investigate systematically antibody responses to WNV infections using a series of overlapping protein fragments spanning all structural proteins of the virus. This technology was used to study the IgG response of humans to linear B-cell epitopes during WNV outbreaks in Italy and Greece in 2010, which were caused by WNV strains belonging to lineages 1 and 2, respectively. Our results identify areas of the structural proteins that are strongly recognized by antibodies.

## Results

**Figure 1:**
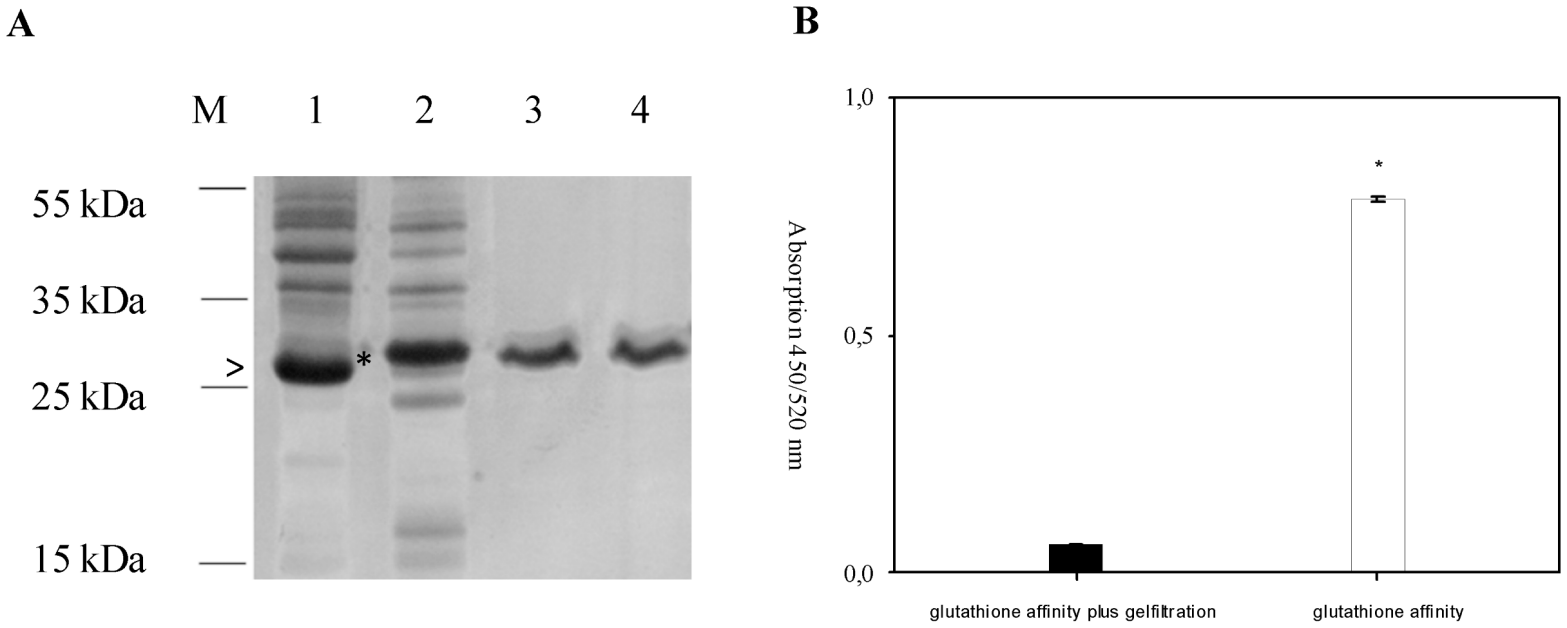
Purification and testing of recombinant proteins. **A**: SDS-PAGE showing crude lysates of protein-expressing bacteria and purification steps. M, size marker; Lane 1, culture expressing GST (arrowhead); lane 2, culture expressing peptide C_41–70_ (asterisk); lane 3, peptide C_41–70_ after glutathione-affinity-purification; lane 4, peptide C_41–70_ after gelfiltration. **B**: ELISA using serum I3 on GST purified only with glutathione sepharose (white column, 500 ng) or additionally with gel filtration chromatography (black column, 2000 ng). Values are the mean of two independent experiments (performed in duplicate), error bars represent the standard deviation. Statistical analysis to evaluate the difference between the two signals was performed by using an unpaired t-test, (asterisks, p<0,001).

**Figure 2:**
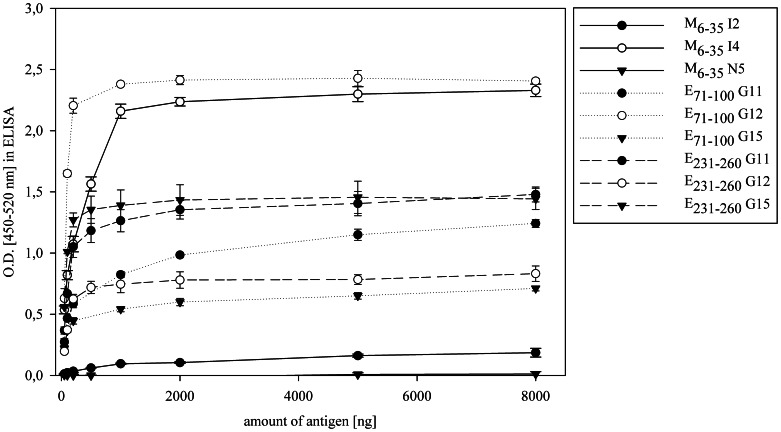
Optimizing antibody binding to selected peptides. ELISA plates were coated with increasing amounts of the indicated peptides fused to GST and incubated with human sera: WNV positive sera I2, I4; G11, G12, G15 and negative serum N5. Values are the mean of two independent experiments (performed in duplicate), error bars represent the standard deviation.

To investigate antibody responses against WNV during European outbreaks, we designed a series of overlapping polypeptide fragments of 30 amino acids in length corresponding to the structural proteins of the Ita09 strain (**Table 1**). These were expressed as GST-fusion proteins in *E.coli* and purified using glutathione affinity chromatography (**Figure 1A**). After analyzing some of the fusion proteins in an ELISA with human sera, considerable background was observed secondary to antibody binding to the GST-only sample (data not shown). Subsequently, size exclusion chromatography was added as a second isolation step, which improved purity of the samples and enhanced the specificity of binding (**Figure 1B**). Next, the optimal concentrations of the fusion proteins in an ELISA was determined by coating increasing amounts of the antigen and analyzing sera from WNV infected individuals. As shown in **Figure 2**, 2 micrograms per well of peptide was sufficient for optimal signal from sera containing either high or low antibody titers. A serum dilution of 1∶100 was chosen because more concentrated samples led to an increase in background binding (data not shown).

**Table 1:** Amino acid sequences of the fragments used in this study (lineage 1 WNV strain Ita09).

Sequence	Peptide name
MSKKPGGPGKSRAVNMLKRGMPRVLSLIGL	C_1–30_
MPRVLSLIGLKRAMLSLIDGKGPIRFVLAL	C_21–50_
KGPIRFVLALLAFFRFTAIAPTRAVLDRWR	C_41–70_
PTRAVLDRWRGVNKQTAMKHLLSFKKELGT	C_61–90_
LLSFKKELGTLTSAINRRSSKQKKRGGKTG	C_81–110_
KQKKRGGKTGIAVMIGLIASVGAVTLSNFQ	C_101–123_/prM_1–7_
VGAVTLSNFQGKVMMTVNATDVTDVITIPT	C_120–123_/prM_1–27_
DVTDVITIPTAAGKNLCIVRAMDVGYMCDD	prM_18–47_
AMDVGYMCDDTITYECPVLSAGNDPEDIDC	prM_38–67_
AGNDPEDIDCWCTKSAVYVRYGRCTKTRHS	prM_58–87_
YGRCTKTRHSRRSRRSLTVQTHGESTLANK	prM_78–92_/M_1–15_
THGESTLANKKGAWMDSTKATRYLVKTESW	M_6–35_
TRYLVKTESWILRNPGYALVAAVIGWMLGS	M_26–55_
AAVIGWMLGSNTMQRVVFVVLLLLVAPAYS	M_46–75_
LLLLVAPAYSFNCLGMSNRDFLEGVSGATW	M_66–75_/E_1–20_
FLEGVSGATWVDLVLEGDSCVTIMSKDKPT	E_11–40_
VTIMSKDKPTIDVKMMNMEAANLAEVRSYC	E_31–60_
ANLAEVRSYCYLATVSDLSTKAACPTMGEA	E_51–80_
KAACPTMGEAHNDKRADPAFVCRQGVVDRG	E_71–100_
VCRQGVVDRGWGNGCGLFGKGSIDTCAKFA	E_91–120_
GSIDTCAKFACSTKATGRTILKENIKYEVA	E_111–140_
LKENIKYEVAIFVHGPTTVESHGNYSTQIG	E_131–160_
SHGNYSTQIGATQAGRFSITPAAPSYTLKL	E_151–180_
PAAPSYTLKLGEYGEVTVDCEPRSGIDTNA	E_171–200_
EPRSGIDTNAYYVMTVGTKTFLVHREWFMD	E_191–220_
FLVHREWFMDLNLPWSSAGSTVWRNRETLM	E_211–240_
TVWRNRETLMEFEEPHATKQSVIALGSQEG	E_231–260_
SVIALGSQEGALHQALAGAIPVEFSSNTVK	E_251–280_
PVEFSSNTVKLTSGHLKCRVKMEKLQLKGT	E_271–300_
KMEKLQLKGTTYGVCSKAFKFLGTPADTGH	E_291–320_
FLGTPADTGHGTVVLELQYTGTDGPCKVPI	E_311–340_
GTDGPCKVPISSVASLNDLTPVGRLVTVNP	E_331–360_
PVGRLVTVNPFVSVATANAKVLIELEPPFG	E_351–380_
VLIELEPPFGDSYIVVGRGEQQINHHWHKS	E_371–400_
QQINHHWHKSGSSIGKAFTTTLKGAQRLAA	E_391–420_
TLKGAQRLAALGDTAWDFGSVGGVFTSVGK	E_411–440_
VGGVFTSVGKAVHQVFGGAFRSLFGGMSWI	E_431–460_
RSLFGGMSWITQGLLGALLLWMGINARDRS	E_451–480_
WMGINARDRSIALTFLAVGGVLLFLSVNVH	E_471–500_
VLLFLSVNVHADTGCAIDISRQELRCGSGV	E_491–501_/NS1_1–18_

**Figure 3:**
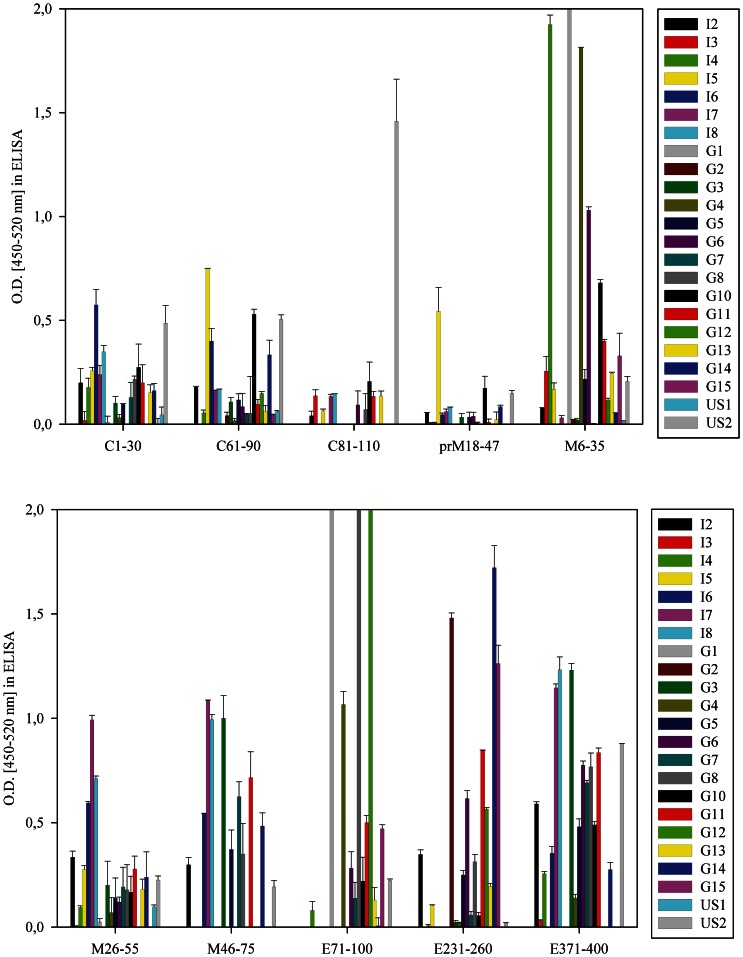
Analysis of the binding property of selected recombinant peptides with human sera in an ELISA. 30-mer peptides spanning the WNV-proteins capsid, prM/M and E, fused to GST, were incubated with human sera from outbreaks in Italy (I1-8), Greece (G1–G15) and USA (US1-2). Those peptides displaying signals with an OD >0,5 are shown (the total number of peptides is shown in Figure S1).Values represent the absorption over cut-off (mean of four negative sera plus two standard deviations) and are derived from at least two independent experiments (performed in duplicate). Error bars represent the standard deviation. The background (binding of the serum to GST) was subtracted.

Sera from WNV-infected humans (with or without clinical symptoms) were obtained from Greece, Italy, and the United States. These sera were tested for reactivity with the panel of fusion proteins by ELISA (**Figure 3** **and Figure S1**). The structural proteome represented by the polypeptides clearly contains regions that were recognized preferentially by antibodies from WNV-infected individuals. The signals were heterogeneous, i.e. only a few fragments were bound strongly by most or all positive sera (e.g. peptide prM_58–87_). However, all WNV-positive sera showed binding over the defined background to at least one fragment, and the vast majority recognized several fragments strongly. In addition, some regions (e.g. the stretch between fragments E_91–120_ and E_171–200_ of the E-protein) displayed marginal reactivity with serum antibodies. The most antigenic linear epitopes appeared to fall within the prM/M-protein, where robust signals were detected in several fragments, most pronounced in M_6–35_. The capsid-protein was recognized as well, but the titers were lower compared to prM/M. Three peptides in the E protein (E_71–100_, E_231–260_ and E_371–400_) resulted in high signals, whereas others (E_11–40_, E_271–300_ or E_311–340_) also were recognized by many sera, albeit with lower intensity. There were no obvious differences between the samples from Italy, Greece, and the USA for the binding pattern to the different peptides or the strength of binding to individual peptides. Fragments C_41–70_, prM_78–92_/M_1–15_, M_66–75_/E_1–20_ and E_131–160_ were excluded from further analysis, as they were recognized strongly by non-immune negative sera.

**Figure 4:**
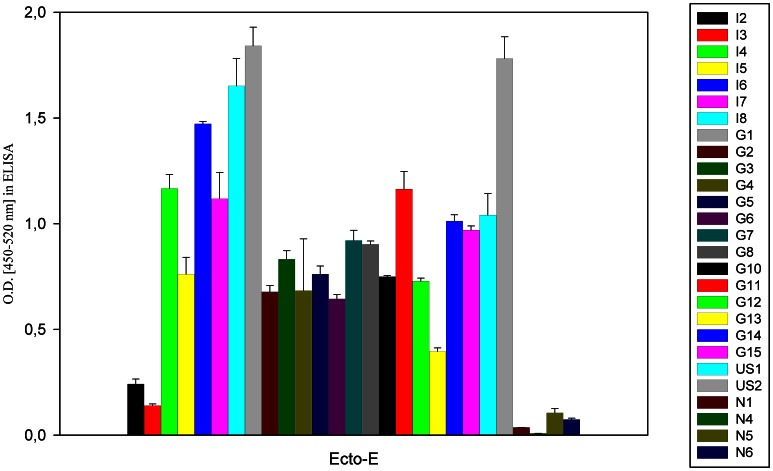
Analysis of binding properties of human sera with recombinant E ectodomain in an ELISA. Sera used in this study were incubated with the recombinant E-ectodomain protein (lineage 1). Mean values of two independent experiments (performed in duplicate) are shown, and error bars represent the standard deviation.

We also assessed whether the heterogeneous antibody responses observed was a feature of the linear epitopes within our peptide assay. We incubated the same sera with recombinantly expressed ectodomain (residues 1–415) of the E protein (New York 1999 strain, lineage 1), which showed the correct tertiary structure folding, as assayed by a panel of conformational-sensitive monoclonal antibodies and X-ray crystallography [29], [30]. Similar to the results obtained with the peptides, antibody titers varied substantially among the different sera (**Figure 4**); hence differences in IgG levels against structural proteins are intrinsic to human WNV infections and not restricted to linear peptide epitopes. Some sera (e.g. US1, G7) showed low or moderate binding to peptides spanning the E protein but bound strongly to the recombinant E-ectodomain, suggesting they recognized more complex structural epitopes, as has been described for some anti-WNV human monoclonal antibodies [31]. Although the values obtained with the two types of antigens are not directly comparable, this suggests that some individuals produce antibodies that recognize structurally discontinuous epitopes on E, which are not displayed by our peptide fragments.

**Figure 5:**
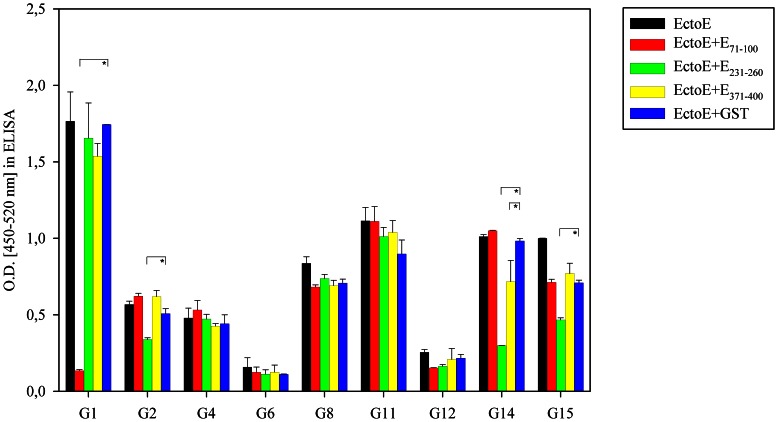
Competition of antibody binding between selected peptides and recombinant E-ectodomain. The indicated sera were pre-incubated with recombinant peptides E_71–100_, E_231–260,_ E_371–400_ (all fused to GST) or GST alone, before they were bound to recombinant E-ectodomain of WNV. Values are derived from at least two independent experiments (performed in duplicate). Error bars represent the standard deviation. Statistical analysis to evaluate the difference between the signals of the competitor peptide and the control competitor (GST alone) were performed by using a Mann-Whitney Rank Sum Test (asterisks, p<0,05).

To investigate the contribution of antibodies against the peptides to the overall antibody response against the E protein, competition experiments were carried out. To this end, sera were incubated with the fusion proteins displaying peptides E_71–100_, E_231–260_ or E_371–400_, before they were tested against the recombinant ectodomain of the E protein. GST-only was used as a control-competitor. As shown in **Figure 5**, a clear competition for binding was observed in some but not all sera, again highlighting the heterogeneity of the human humoral responses to WNV. In serum G1, for example, the reactivity to the E ectodomain was almost completely lost after pre-incubation with peptide E_71–100_, indicating that this linear epitope is a major target of the anti-E response in that particular patient. Similar results were obtained for E_231–260_ with serum G14 and, although less prominent, with sera G2 and G15.

## Discussion

Information on the immune responses of humans against newly emerging European strains of WNV is still limited, especially when compared to data available after infections with North American strains. The WNV epidemic in Europe differs from that in North America in several aspects, such as pathogenicity in birds and the vector ecology [32]. Also different WNV strains from distinct genetic lineages are responsible for the outbreaks in Europe. For infections with the WNV isolates from North America, immunodominant peptides eliciting T cell responses in humans have been determined [21], [33]. Although epitopes for monoclonal antibody responses have been mapped [22], [29], no systematic peptide mapping has been performed with sera from infected humans. In this study, we investigated antibody responses to the structural proteins of WNV during European outbreaks, which occurred in Greece and Italy in 2010. By screening overlapping 30-mer peptides with human sera, areas in the structural proteome were identified as targets of IgG antibodies, indicating the presence of several linear B cell epitopes within these peptides.

One major observation was that the antibody profiles against the peptides corresponding to the structural proteins differed substantially between individuals. This heterogeneity of binding can best be explained by the variation in the human immune response to different epitopes and/or differences in sampling as serum was collected at different time points after symptoms onset [34]. Heterogeneity in human antibody responses against WNV infections also was recently demonstrated by [35] who tested four different sera on recombinant WNV proteins and obtained four unique binding patterns. While differences in the HLA class I- and class II-background are associated with protection or susceptibility to neuroinvasive WNV disease [36], a direct correlation between severity of infection and the immune response to specific viral peptides could not be identified in a screen for CD8^+^ T cell-epitopes [21]. Analogously, we could not define a correlation between IgG responses to specific areas of the WNV-structural proteome and clinical symptoms (data not shown).

**Figure 6:**
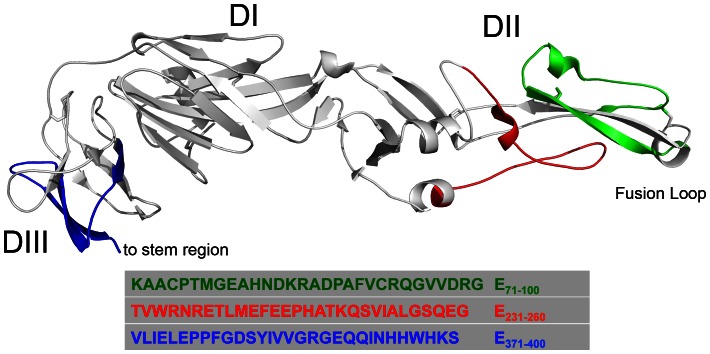
Structure of the E-ectodomain of WNV. The sequences corresponding to the peptides E_71–100_, E_231–260_ and E_371–400_ are highlighted in color. DI, DII and DIII indicate the respective domains.

Based on the results, the immunodominant peptides were distributed throughout in the capsid (peptide C_61–90_), prM/M (peptides M_6–35_ to M_46–75_), and E (peptides E_71–100_ and E_231–260_, which are in domains I and II, and E_371–400_ in domain III) proteins. prM/M protein was the most immunogenic in our analysis. Every infected individual in the study developed antibodies against one or several fragments of prM/M, making it a valuable protein for serologic diagnosis and thus an important constituent of VLP-based test systems [37]. The immunogenicity of prM protein in the context of human infection has been highlighted recently in studies with Dengue virus, a related flavivirus [38]–[40]. The region displayed by peptides M_6–35_ and M_26–55_ was shown to elicit Dengue-neutralizing antibodies in mice [41]. Within the E protein, peptides E_71–100_, E_231–260_ and E_371–400_ appeared immunodominant, and the three sequences were mapped onto the structure of the E ectodomain (**Figure 6**). Only fragment E_371–400_ is situated in domain III (encompassing parts of the lateral ridge FG-loop), where dominant epitopes for neutralizing antibodies in mice are found [42]–[44]. This data is consistent with studies suggesting that the human antibody response against E is skewed away from DIII and towards less-neutralizing epitopes in domains I and II [22], [29]. Indeed, peptide E_71–100_, which is located near or within the fusion loop in domain II of the E protein, was one of the dominant peptides in the present analysis; however, fusion loop reactive monoclonal antibodies tend to have poorly neutralizing activity against lineage 1 strains of WNV [45], [46].

The results obtained with the binding to individual peptides were confirmed by competition studies between peptides and the recombinant E-ectodomain. In some sera (G1, G2, G15), which showed strong binding to only one peptide in the E-protein (**Figure 3**), the signal for the E-ectodomain was reduced after pre-incubation with this peptide. As G1, G2 and G15 recognize the folded E-ectodomain well (**Figure 4**), these results indicate that the linear sequences corresponding to E_71–100_, E_231–260_ and E_371–400_ are accessible for antibody binding. Using sera that showed binding to multiple E-peptides in the screen, the competition with a single peptide did not significantly change binding to the E-ectodomain (e.g. G8, G11 or G12). However, serum G4 only binds strongly to E_71–100_, yet there was no competition with this peptide. Together with the finding that in sera G2, G14 and G15 there was still a considerable signal left after competition, the data from the competition tests confirm that antibodies against tertiary structural epitopes on the E-ectodomain, not displayed by our peptides, are generated preferentially during infection in some individuals.

By using sera from a WNV outbreak in Israel, [47] described a linear B-cell epitope in the E protein, which reacted with some, but not all WNV positive sera. That epitope, located within E_351–380_, only was recognized marginally by the sera from our study. Differences in the infecting strain (e.g. the Ita09 strain contains the point mutation A369S within the epitope), in the B cell response by different populations, or in the methodology (e.g. the EP15 epitope of [47] was used as a tandem peptide fused to a phage protein) might contribute to the disparity in findings.

Somewhat surprisingly, all humans did not develop robust antibody responses against the recombinant E protein. Serum I3 had low levels of antibodies against the E protein ectodomain or peptides, although a clear response against prM peptide M_6–35_ was observed. One caveat to this analysis is that antibodies by patient I3 could have been generated against E protein epitopes that are present exclusively on the icosahedral surface of the intact virion, which are absent from isolated recombinant E protein or peptides. Indeed, such quaternary epitopes that localize to the hinge region between domains I and II on the E protein have been described for human monoclonal antibodies against WNV and DENV [31], [48]–[50]. Antibodies against the capsid mainly reflect immune responses against disrupted virions or apoptotic or necrotic infected cells in which intracellular contents are released. These are unlikely to have a protective phenotype, except supporting antigen presentation by antigen presenting cells.

In summary, we show that several peptides of the capsid, the prM/M, and the E proteins of WNV are recognized by IgG-antibodies from humans infected with newly emerging European strains of WNV. All WNV-positive sera bound strongly to at least one peptide (corresponding to the lineage 1 amino acid sequence) and no clear differences were observed between sera obtained from patients from Greece, Italy or the USA that were infected with lineage 2 or 1 strains. However, the data reveal substantial differences between individual sera in the patterns of the proteins and domains recognized, highlighting the heterogeneity of the human humoral immune response against WNV, which should be considered during the development of specific diagnostic tests.

## Materials and Methods

### Protein Expression and Purification

The sequence coding for amino acids 1–810 of the lineage 1 WNV strain “Ita09” (NCBI Acc#. GU011992) was separated into 40 clones, each coding for 30 amino acid long peptides with an overlap of 10 amino acids on both sides. The length of overlapping 30 amino acids was chosen to optimally detect long linear epitopes in combination with fine-mapping of the humoral response. The DNA fragments were produced in the bacterial expression vector pEXP1 by ATG: biosynthetics (Freiburg, Germany) and contain the protein glutathione-S-transferase (GST) from *Schistosoma japonicum* fused to the N-terminus. Plasmids were transformed into the *E.coli* BL21 strain. Colonies were selected from kanamycin-containing agar plates, incubated overnight in 20 mL of LB media at 37°C, and protein expression was induced by IPTG (final concentration, 1 mM). After 16 h of induction, the bacteria were harvested by spinning for 20 min at 5.000×g at 4°C. The bacterial cell pellets were resuspended in lysis buffer (20 mM Tris pH 7,4, 200 mM NaCl, 1 mM EDTA). Lysates were obtained after sonication followed by three freeze-thaw cycles. Subsequently, the lysates were clarified by centrifugation (20 min at 15,000×g at 4°C). The supernatant was isolated and stored at –80°C until usage. Clarified lysates containing the GST-fusion proteins were purified by Glutathione-sepharose chromatography (Qiagen, Germany) and analysed by SDS-PAGE. A second purification using size exclusion chromatography (Sepharose 6FF 26/10, GE Healthcare) was performed to reduce background binding. The WNV E protein (ectodomain, amino acids 1–415 of New York 1999 strain) was expressed in *E. coli* and purified as described previously [29].

### Sera

Serum samples from confirmed WNV-infections were obtained during outbreaks in Italy and Greece in 2010. The Italian samples (University of Padova, Italy) were derived from seroprevalence studies, blood donors or patients with West Nile neuroinvasive disease. The Greek samples (University of Thessaloniki, Greece) were from patients with neuroinvasive disease, taken during the acute phase of illness (3–17 days). In addition, two samples were obtained from Seracare (USA). None of the patients were vaccinated against other flaviviruses or had a recent travel history to other countries endemic for WNV. Patients were 32–83 years old, and only one was born before the Dengue-outbreak in Greece in 1927 [18]. WNV-negative samples were from each source and from the University of Leipzig (Germany). The study was approved by the Padova University Hospital ethics committee and all persons provided written consent.

### Antibody – Peptide Binding Analysis by ELISA

Nunc polysorb plates (Thermo Scientific, Germany) were coated in duplicate with 2 micrograms of GST-fusion proteins or with 100 nanograms of recombinant E ectodomain protein (in coating buffer (15 mM Na_2_CO_3_, 35 mM NaHCO_3_ pH 9.6)) per well and were incubated over night at 4°C with gentle agitation. The plates were then washed three times with 350 microliters per well of PBS/Tween (0.05%), followed by blocking with 5% non-fat dry milk powder (200 microliters per well) for 2 h at room temperature (RT). After a second wash step, the human sera (dilution 1∶100 in 5% non-fat dry milk powder, 100 microliters per well) were incubated for 1.5 h at RT. The sera were removed by a third wash step and 100 microliters of the secondary antibody (1∶5000 diluted HRP-conjugated Goat-anti-Human IgG, Fisher Scientific) was added for 1 h at RT. After washing, the TMB-substrate (BioLegend, Germany) was added to the wells and the plate was incubated for 30 min at RT in darkness. To stop the reaction, 1 M H_2_SO_4_ was added, followed by measurement at 450 nm and 520 nm (reference wavelength) in an ELISA Reader (Infiniti M200, Tecan). The cut-off-values were defined for each individual antigen by calculating the mean of four negative sera (derived from Italy, Greece and Germany) and adding two standard deviations. Competition ELISA was performed as described above, except that 50 nanograms recombinant E ectodomain was coated and the sera were pre-incubated with 4 micrograms GST-fusion protein or GST for 45 min at RT.

## Supporting Information

Figure S1 **Analysis of the binding property of all recombinant peptides used in the study with human sera in an ELISA.** 30-mer peptides spanning the WNV-proteins capsid, prM/M and E and a small part of NS1, fused to GST, were incubated with human sera from outbreaks in Italy (I1-8), Greece (G1–G15) and USA (US1-2). Values represent the absorption over cut-off (mean of four negative sera plus two standard deviations, indicated below the peptide names) and are derived from at least two independent experiments (performed in duplicate). Error bars represent the standard deviation. The background (binding of the serum to GST) was subtracted.
(PDF)
