# Supplementary figures and images for: Antibody Responses in Humans Infected with Newly Emerging Strains of West Nile Virus in Europe
Source: PLoS One. 2013 Jun 12;8(6):e66507. doi: 10.1371/journal.pone.0066507 (PMC3680493; doi:10.1371/journal.pone.0066507)

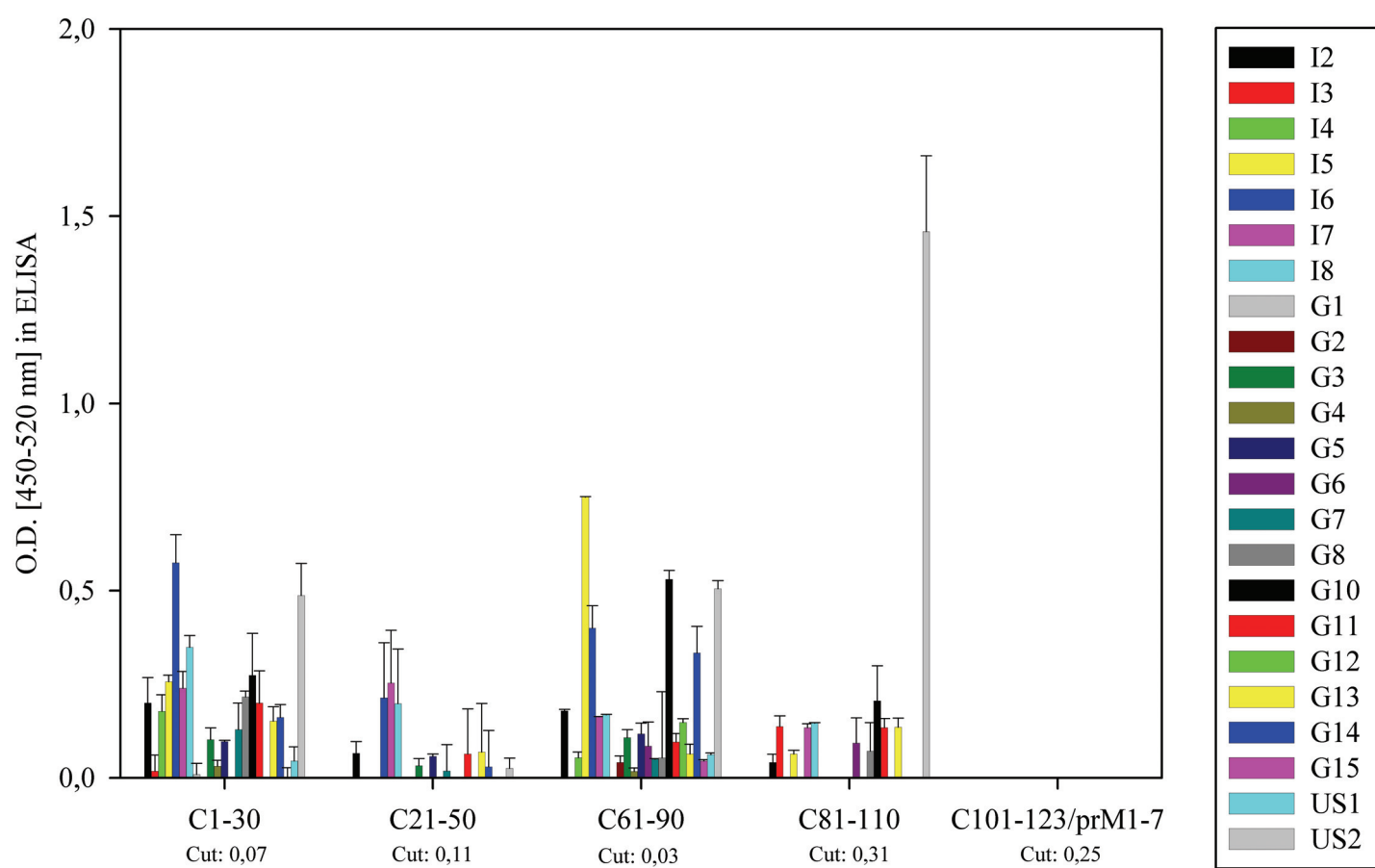

Figure S1

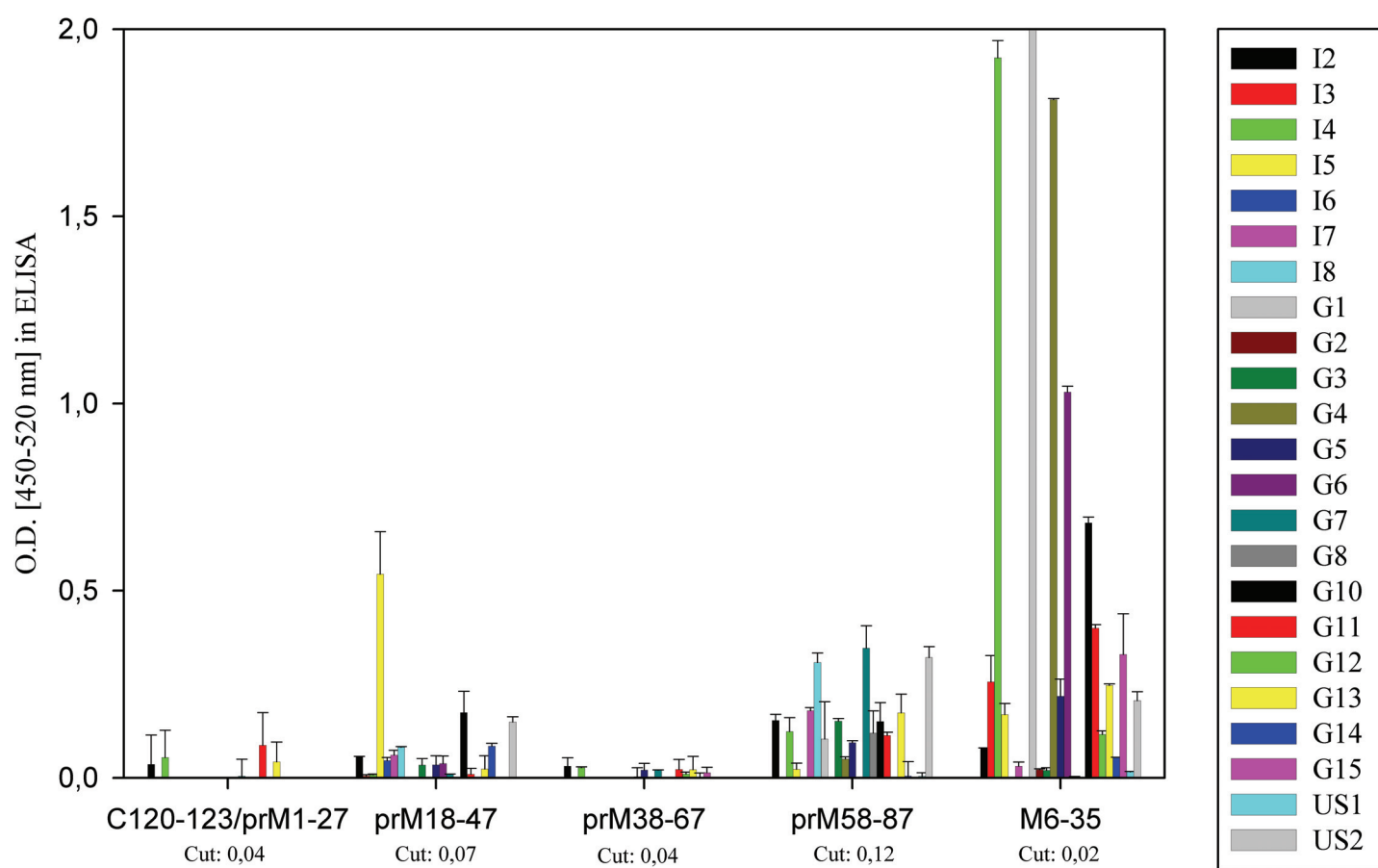

Figure S1

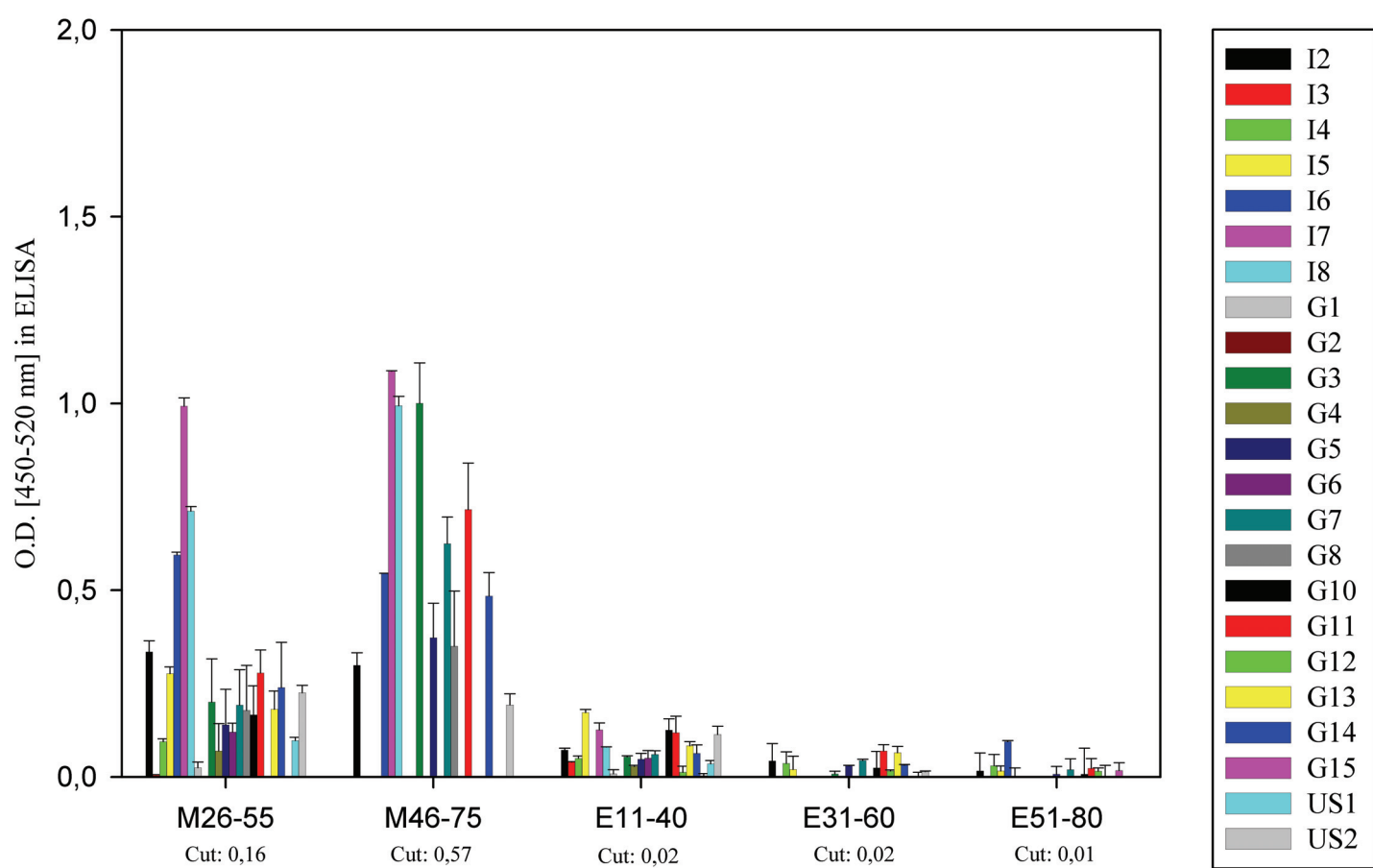

Figure S1

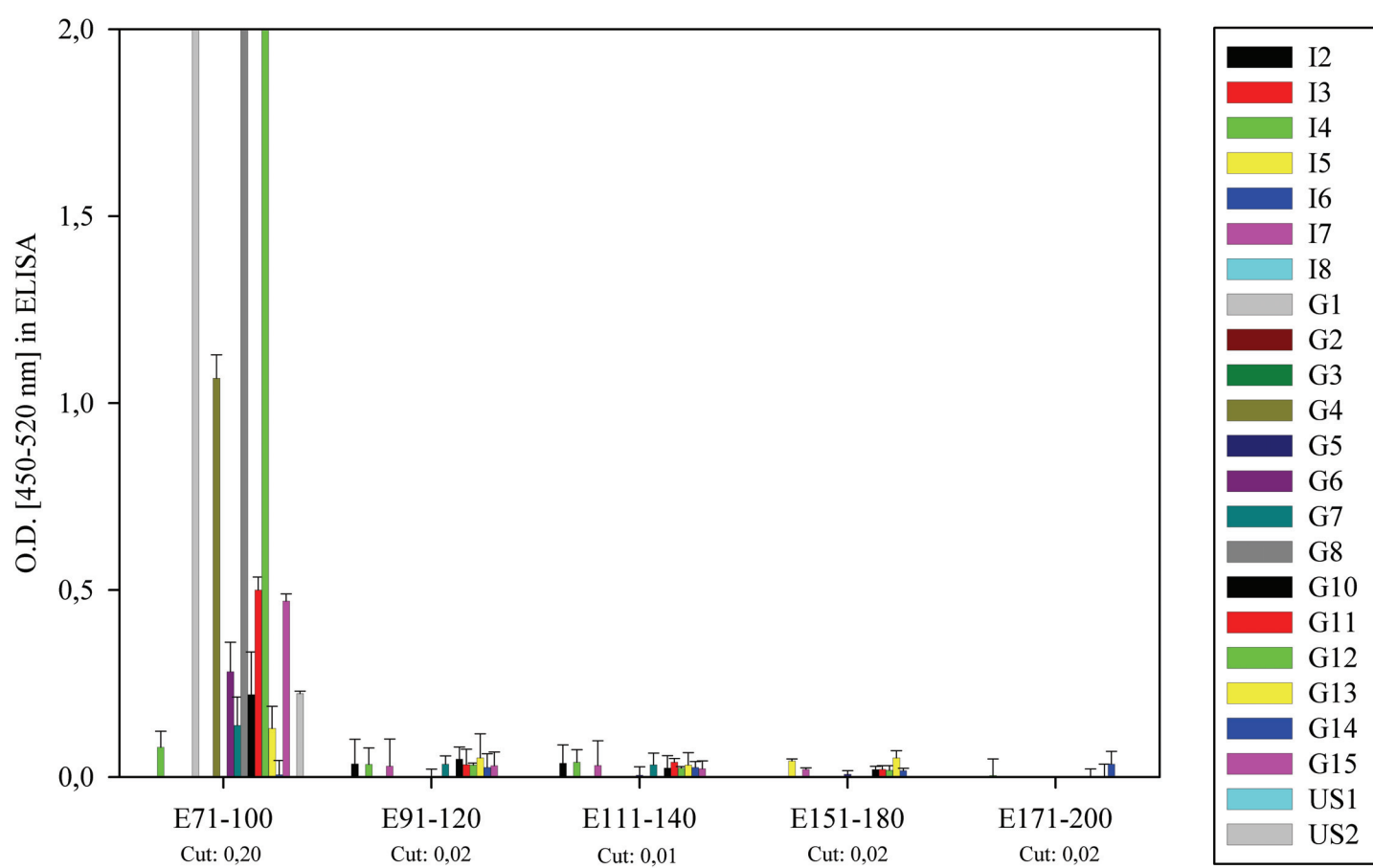

Figure S1

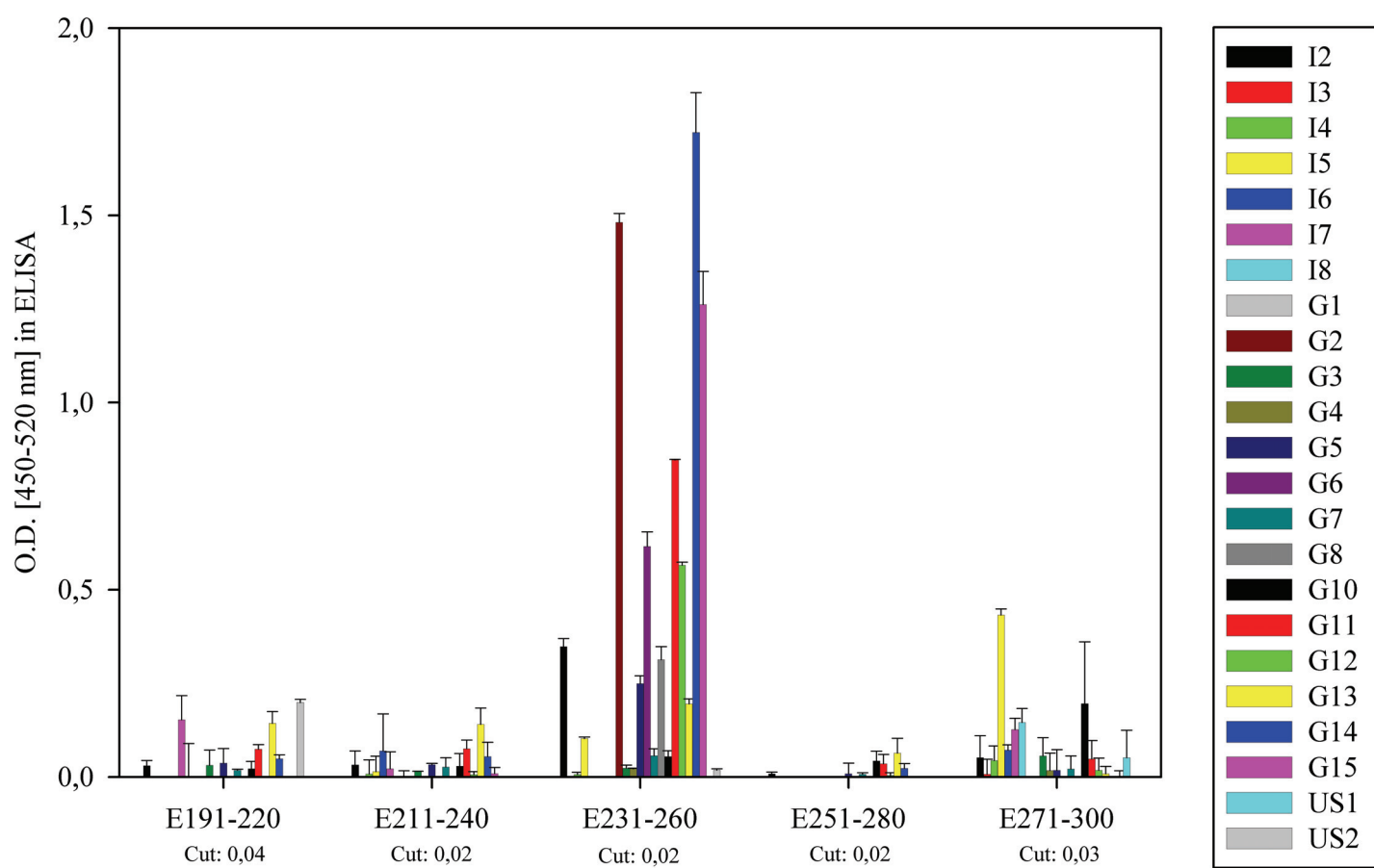

Figure S1

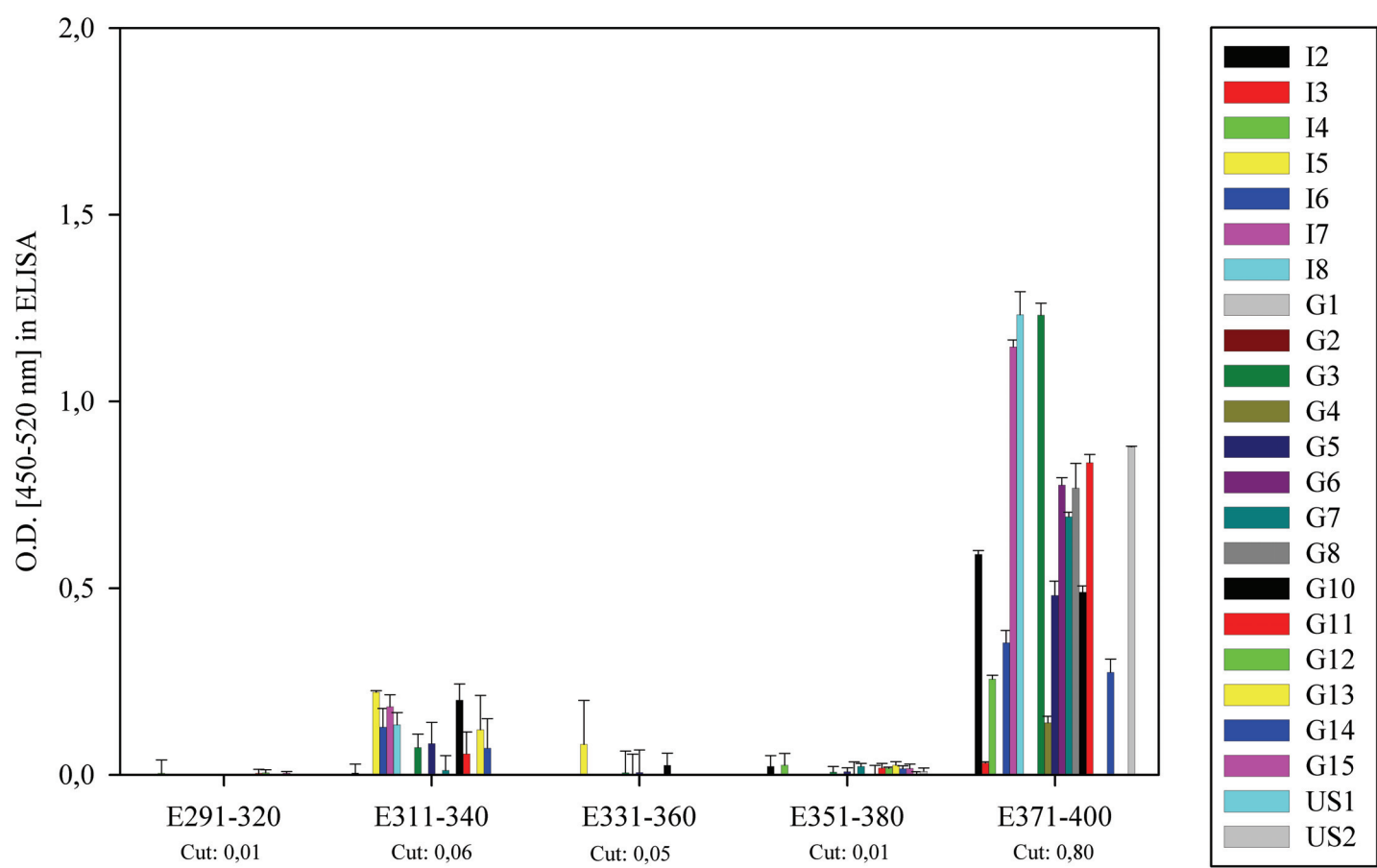

Figure S1

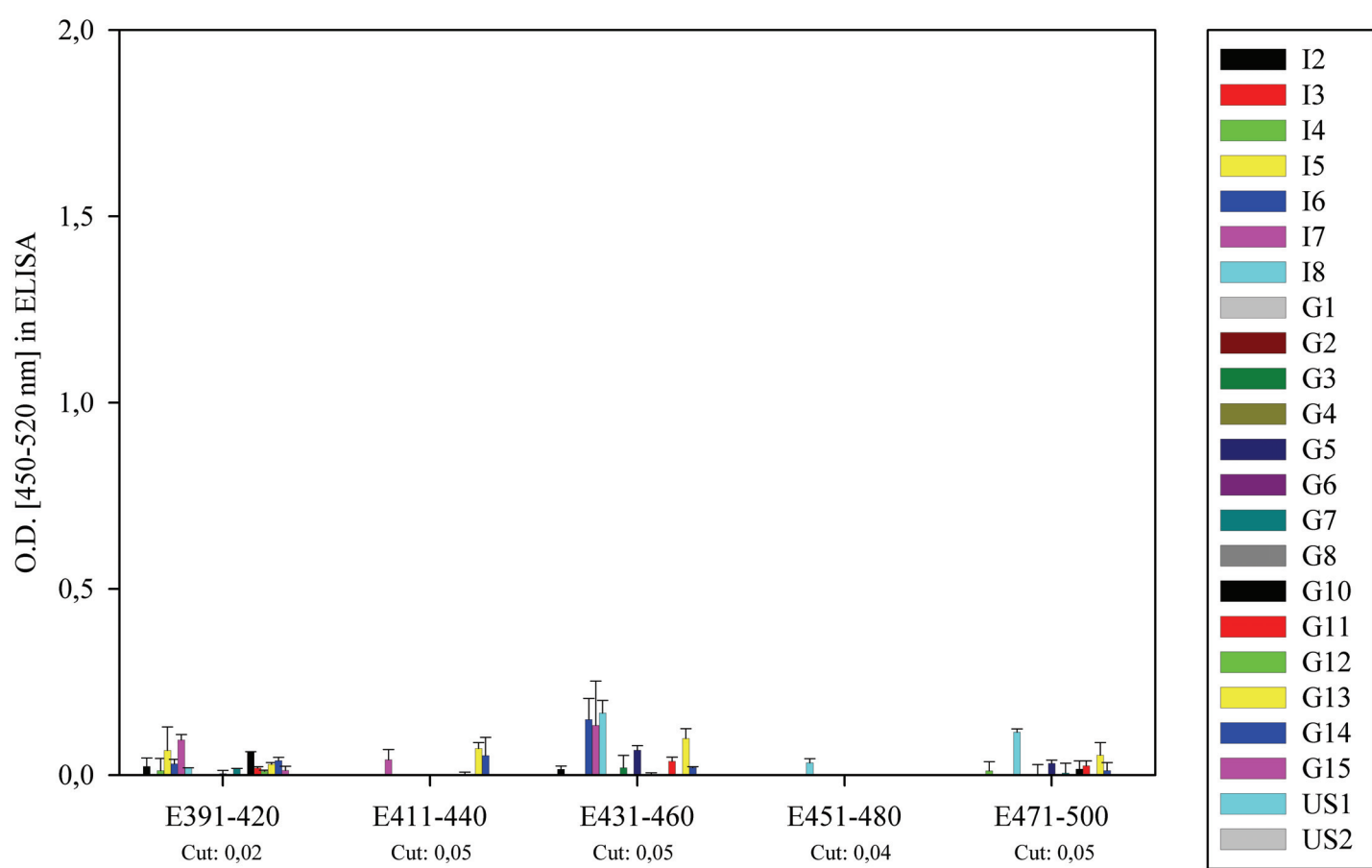

Figure S1

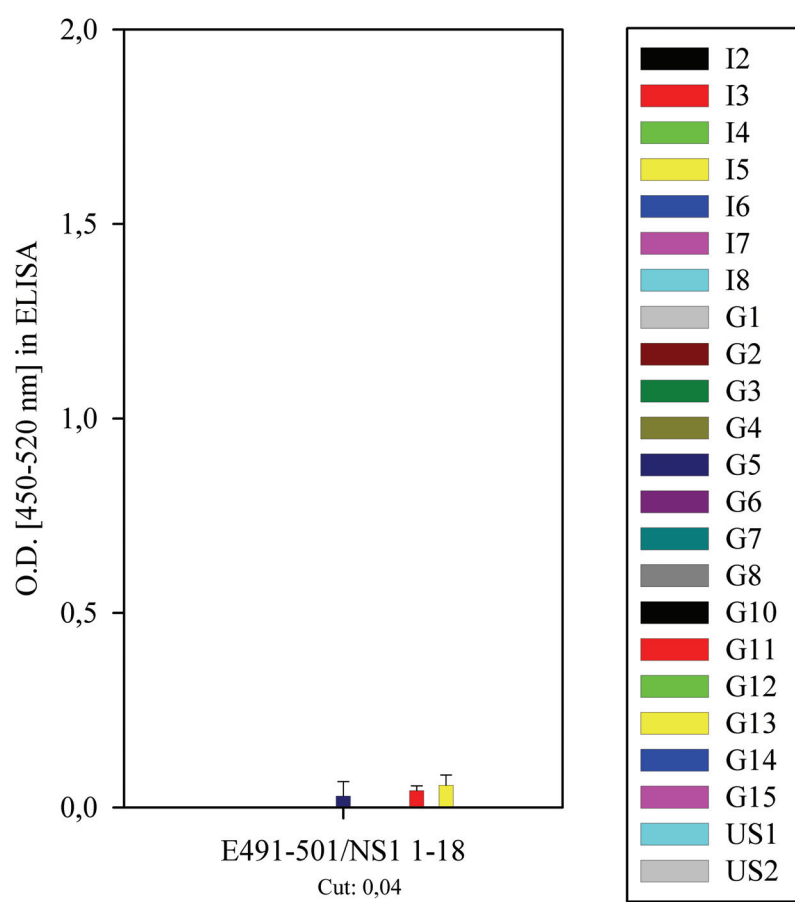

Figure S1

Supplement: Figure S1 — Analysis of the binding property of all recombinant peptides used in the study with human sera in an ELISA. 30-mer peptides spanning the WNV-proteins capsid, prM/M and E and a small part of NS1, fused to GST, were incubated with human sera from outbreaks in Italy (I1-8), Greece (G1–G15) and USA (US1-2). Values represent the absorption over cut-off (mean of four negative sera plus two standard deviations, indicated below the peptide names) and are derived from at least two independent experiments (performed in duplicate). Error bars represent the standard deviation. The background (binding of the serum to GST) was subtracted. (PDF) [file pone.0066507.s001.pdf]
